# Movement Behaviour of Traditionally Managed Cattle in the Eastern Province of Zambia Captured Using Two-Dimensional Motion Sensors

**DOI:** 10.1371/journal.pone.0138125

**Published:** 2015-09-14

**Authors:** Caesar H. Lubaba, Arata Hidano, Susan C. Welburn, Crawford W. Revie, Mark C. Eisler

**Affiliations:** 1 Division of Infection and Pathway Medicine, Centre for Infectious Diseases, School of Biomedical Sciences, College of Medicine and Veterinary Medicine, The University of Edinburgh, Edinburgh, United Kingdom; 2 Centre for Veterinary Epidemiological Research, Atlantic Veterinary College, University of Prince Edward Island, Charlottetown, Canada; 3 School of Veterinary Sciences, University of Bristol, Bristol, United Kingdom; ETH Zurich, SWITZERLAND

## Abstract

Two-dimensional motion sensors use electronic accelerometers to record the lying, standing and walking activity of cattle. Movement behaviour data collected automatically using these sensors over prolonged periods of time could be of use to stakeholders making management and disease control decisions in rural sub-Saharan Africa leading to potential improvements in animal health and production. Motion sensors were used in this study with the aim of monitoring and quantifying the movement behaviour of traditionally managed Angoni cattle in Petauke District in the Eastern Province of Zambia. This study was designed to assess whether motion sensors were suitable for use on traditionally managed cattle in two veterinary camps in Petauke District in the Eastern Province of Zambia. In each veterinary camp, twenty cattle were selected for study. Each animal had a motion sensor placed on its hind leg to continuously measure and record its movement behaviour over a two week period. Analysing the sensor data using principal components analysis (PCA) revealed that the majority of variability in behaviour among studied cattle could be attributed to their behaviour at night and in the morning. The behaviour at night was markedly different between veterinary camps; while differences in the morning appeared to reflect varying behaviour across all animals. The study results validate the use of such motion sensors in the chosen setting and highlight the importance of appropriate data summarisation techniques to adequately describe and compare animal movement behaviours if association to other factors, such as location, breed or health status are to be assessed.

## Introduction

Motion sensors have been found to be relatively accurate when compared to video analysis for classifying cattle behaviour into one of three activities: standing, active or lying down [1]. In addition, motion sensors offer the potential to remotely monitor animal movement behaviour and to obtain an objective and non-invasive measure of activity in an automated manner [2, 3]. If these technologies could be utilised in rural settings, they could provide useful information under conditions where diseases causing anaemia or lethargy might be expected to affect animal behaviour [4–9]. Thus, it is interesting to examine the performance of motion sensors in these settings and understand the conditions under which their use is appropriate. Improved approaches to characterising animal movement behaviour will allow for a clearer assessment of their relationship to health status and should facilitate the more timely detection of disorders in animals. Several studies have been conducted to quantify the association between cattle health status and their behaviour in large and enclosed production settings [10, 11], although there is to date little information on how cattle behaviour monitoring technology might be applied to smaller-scale or traditional production conditions in tropical countries.

Our study objectives were to examine whether motion sensors could be successfully used in a rural context in Eastern Zambia and if so to demonstrate analytical methods to effectively summarise the behavioural characteristics of animals from the extensive quantity of recorded data.

## Materials and Methods

### Ethical Statement

Commercially available motion sensors (IceTag, Ice Robotics, Edinburgh, UK) specifically designed for use in cattle were temporarily attached to the hind legs of the selected cattle at the metatarsus proximal to the fetlock joint using a comfortable proprietary Velcro strap specifically supplied by the manufacture for this purpose. The movement data were collected over 14 days and downloaded to a computer on day 7 and day 14 after removal of the motion sensors from the animals. Animals remained under the usual management routines of their owners in the local community before, during and after the study and hence 'method of sacrifice’ is not applicable.

Authority for all sampling procedures and experimental manipulations were obtained from the Government of Zambia through the Director, DVLD, Ministry of Agriculture and Cooperatives. This was standard approved practice in Zambia at the time the field work was carried out in 2006/2007, which preceded by the Zambian parliament’s enactment of the National Health Research Act of 2013 that provides for the regulation of all health related research including veterinary research. All sampling procedures and/or experimental manipulations were reviewed and specifically approved by the Director, DVLD as stated above. Local area leaders were also consulted prior to attachment of motion sensors. The purpose of the study and procedures to be carried out were explained to farmers and informed consent was provided orally.

### Study area

For veterinary administration purposes, the DVLD has its headquarters in Lusaka, the capital city of Zambia. The country has 10 provinces which are divided into districts. The districts are further subdivided into veterinary “camps”, which are the lowest administrative unit for the DVLD. The two study sites in this study were in the Kasero and Makale veterinary camps of Petauke District in the Eastern Province of Zambia. Kasero lies in an area of plateau to the south of Petauke district, while Makale is in a more northerly location in the Luangwa Valley area near Luangwa national park. The two veterinary camps were selected from others in the area as they are known to have high prevalence of trypanosomiasis and theileriosis based on previous surveys conducted in the area [12, 13]. The two areas were geographically well dispersed across Petauke District, being approximately one hundred kilometres apart and were also accessible by vehicle throughout the year. At the time of the study there were 80 and 41 households that had at least one head of cattle in Kasero and Makale, respectively. Mean numbers of cattle kept per household were 9.4 in Kasero and 5.4 in Makale. Cattle in both camps were kept confined in kraals during the night and were left to graze in communal grazing areas near the village during the day. The study was carried out during the rainy season, in the months of December 2006 and January 2007 and animals did not have to travel far for feed or water which were abundant.

### Motion sensors and animals

IceTag activity monitors are motion sensors designed specifically for use on animals [14] and were used in this study. These sensors (IceTag version 2.004) use a two-dimensional electronic accelerometer to automatically determine the number of steps taken and the percentage of time that an animal spends lying down, standing or active. Data is captured 8 times per second and algorithms are used to determine the average percentage of time allocated to each of these behaviours [2, 14]. The animals used in the study were Angoni cattle which are short horned Zebus found in the Eastern Province of Zambia and adjoining areas of Malawi and Mozambique. These animals are well adapted to a wide range of conditions and are known for their ability to produce a calf every year under low input traditional husbandry systems [15]. The Angoni cattle are highly susceptible to diseases such as theileriosis and trypanosomiasis [16].

### Study design: Cattle selection and pairing

A total of 432 Angoni cattle were presented by farmers in both Makale (*n* = 221) and Kasero (*n* = 211) Veterinary Camps. A total of 10 pairs of animals (i.e. 20 cattle) were selected in each vet camp, excluding draft animals. Cattle in the same pair had the same sex, were close in age, and were from the same kraal and hence co-grazing group. Motion sensors were exchanged between cattle in these pairs as described below.

### Attachment of motion sensors and data collection

The motion sensors were attached to the hind legs of the selected cattle at the metatarsus, proximal to the fetlock joint using a Velcro strap. The attachment and removal of motion sensors was carried out while the animals were restrained at handling facilities which are used for routine animal health practice by local veterinarians. The movement data were collected over 14 days and downloaded to a portable computer on Day 7 and Day 14 to avoid losing all data in the event of the loss of, or damage to, a motion sensor unit. A cross-over design was used to increase the chances of detecting any abnormally functioning motion sensors, in which the sensors were exchanged between cattle in the same pair on Day 7. This design should better facilitate the detection of erroneous data being generated, such as in the event of there being a faulty motion sensor unit.

### Data analysis

#### Data conversion

The raw motion sensor data was first summarised by hour which led each animal to have maximum of 24 summary observations per day (data available in S1 Dataset). Note that several days have less than 24 observations because behaviour was not always recorded throughout the whole day at the start, cross-over and end of study (i.e. on Days 1, 7, and 14). Each observation consisted of four variables; the proportion of time an animal spent standing (*S*_*t*_), active (*A*_*t*_) or lying down (*L*_*t*_) and the number of steps taken (*P*_*t*_) during a given hour *t*. The data was further converted to a matrix with each animal having 96 variables (24 hours × 4 variables of behaviour), to represent the average proportion of time spent standing (*S*_avg_), active (*A*_avg_) and lying down (*L*_avg_) or the average number of steps taken (*P*_avg_) during a given hour *t* over the study period of 14 days.

#### Statistical analysis

The correlation between the number of steps taken (*P*_*t*_) and the proportion of time active (*A*_*t*_) during a given hour was investigated. A scatter plot of observations for every animal in each hour of the study period was generated and Pearson’s correlation coefficient (*r*) was obtained. Principal components analysis (PCA) was used to detect patterns present in the data, to identify the main sources of variability, and to determine effective data dimensionality. PCA provides a method to reduce the number of variables in a multivariate dataset while maintaining as much of the original information as possible in a smaller set of variables that account for most of the variance in the original data [17]. For each observation *k*, PCA generates a set of new components *Z*_1_, *Z*_2_, *Z*_3_…*Z*_N_ based on the original *N* variables *X*_1_, *X*_2_, *X*_3_…*X*_N_ as follows.

$$Z_{nk}=\sum_{j=1}^{N}a_{nj}(X_{jk}-\bar{X_{j})}$$

where $\bar{X_{j}}$ is the average of variable *X*_*j*_ across all observations, and *a*_*nj*_ is the calculated loading value (coefficient) of variable *X*_*j*_ at components *Z*_*n*_. The value for *Z*_*n*_ is referred as score of the *n* ^th^ Principal Component (PC_*n*_). In this study, the original variables are the mean percentages for each behaviour variable (*S*_avg_, *A*_avg_ and *L*_avg_) resulting in *S*_*nk*_ which represented scores of the PC_*n*_ for animal *k*. PCs were calculated for the covariance matrix of the data since all variables were expressed in the same unit (i.e. percentage). The absolute value of loadings indicates the magnitude of the effect of each original variable on a given PC. The sign of the loading also indicates whether each variable is positively or negatively correlated with the PC. Therefore, it is useful to investigate the loadings of each variable on the primary PCs to understand the relationship among variables, and between the variables and these PCs.

The intraclass correlation coefficient (ICC) of scores on each PC (*S*_*nk*_) was calculated using analysis of variance (ANOVA) to examine the degree of between-vet camp and between-animal variations in each PC. The one-sided *F* test was used to examine whether the ICC was different from zero. Logistic regression was conducted to obtain the combination of PCs that best separated the animals according to their vet camp of origin using the minimum number of PCs. To validate the use of percentages for each variable averaged over the study period (*S*_avg_, *A*_avg_ and *L*_avg_), *S*_*nki*_ representing scores on PC_*n*_ for animal *k* on day *i* were calculated using loadings for each variable at each PC and the percentage of time associated with each variable *S*_*t*_, *A*_*t*_, and *L*_*t*_. ICCs for *S*_*nki*_ were also obtained using ANOVA to examine how the level of within-animal variation for each PC compared to the between-animal variation. All data analyses were conducted using RapidMiner 5.0 (Copyright 2001–2010 by Rapid-I and contributors), R ver. 3.1.2 (R Core Team, 2014), and Stata 12 ver.1 (StataCorp, College Station, TX, USA).

## Results

### Correlation between step counts and percentage of walking activity per hour

**Fig 1:**
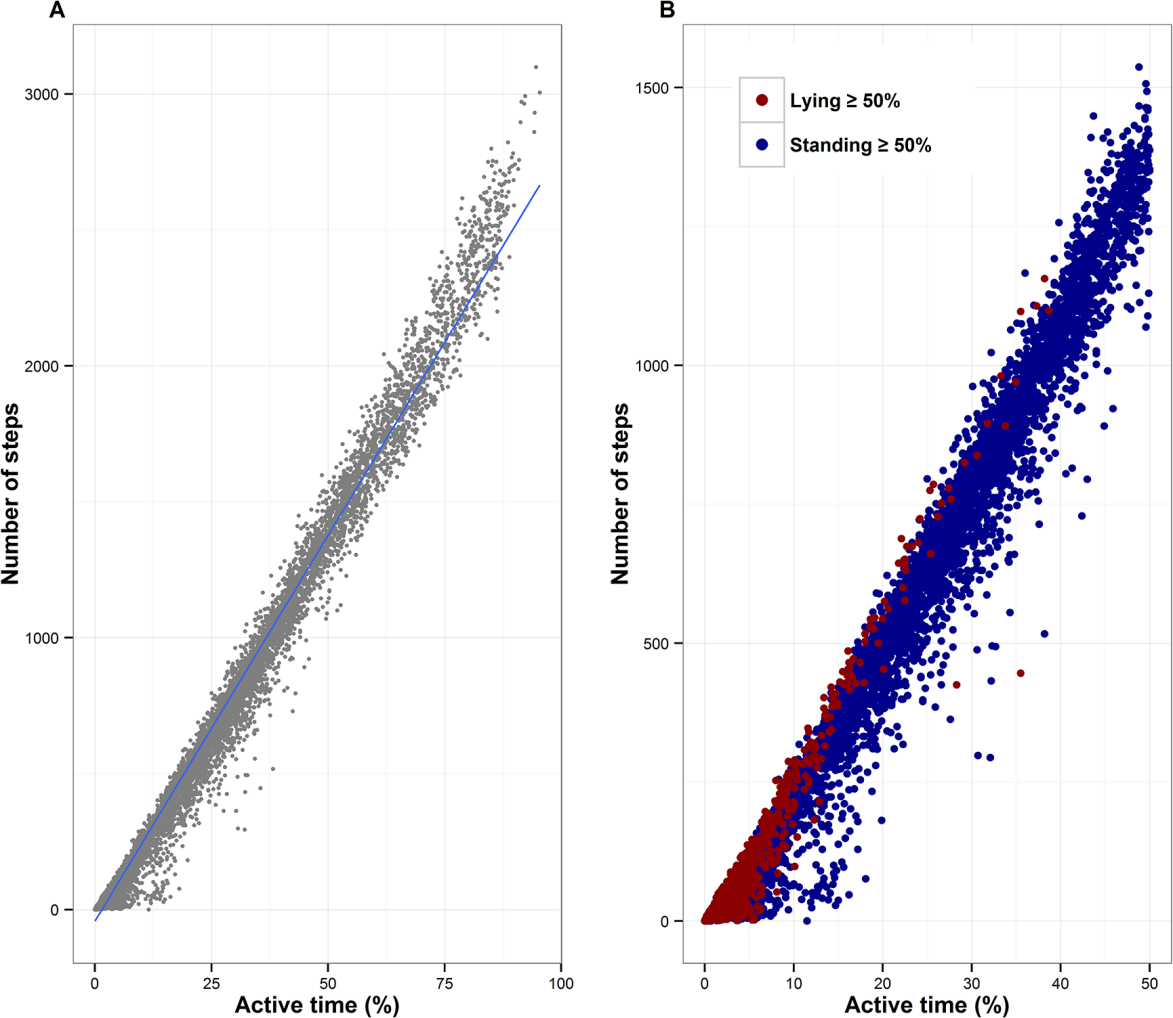
Correlation between steps and percentage of time involved in active behaviour in a given hour. Scatter plot illustrating the association between the number of steps taken and the proportion of time spent in active behaviour for 40 cattle between December 2006 and January 2007 in the Kasero and Makale veterinary camps of Petauke District in the Eastern Province of Zambia. [A] Scatter plot and fitted linear trend (Pearson’s correlation coefficient = 0.994) based on all 24 hours (*n* = 12,745). [B] Scatter plots for only those hours where cattle spent 50% or more time in standing (shown in blue circles) or lying (red circles) behaviour. (Note that the scales on the x and y-axis in [B] are halved compared to those in [A] because the maximum proportion of time that can be spent in active behaviour is 50%.)

Motion sensor data output comprised four variables, three of which were the proportions of time that an animal engaged in a particular activity. For any given hour, the motion sensor algorithm could determine the proportion of time an animal spent standing, active or lying down. The final variable in the motion sensor data output represented the number of steps that an animal took during the hour of interest. Used motion sensors successfully captured cattle behaviour without any missing or faulty data. Fig 1A shows the relationship between the number of steps taken by individual animals in each hour and the corresponding proportion of time active for all animals over the two week study period. There was a very high correlation observed between the time active in a given hour and the number of steps taken during that same hour (*r* = 0.994, p < 0.001). On this basis, the variable summarising the proportion of time active was considered sufficient to allow full use of the data without the need to include the highly correlated ‘steps’ variable. Despite the high correlation coefficient between steps taken and percentage of time spent in active behaviour, some points appeared to demonstrate poor agreement. Further investigation was undertaken to attempt to uncover the factors associated with these outliers. Fig 1B illustrates the paired observations of steps and percentage of active behaviour for all cases where animals spent at least 50% of a given hour either standing or lying down. This observation indicates that the majority of the poorly fitting points were associated with hours during which animals spent the majority of their time standing rather than lying down.

### Principal Components Analysis

**Fig 2:**
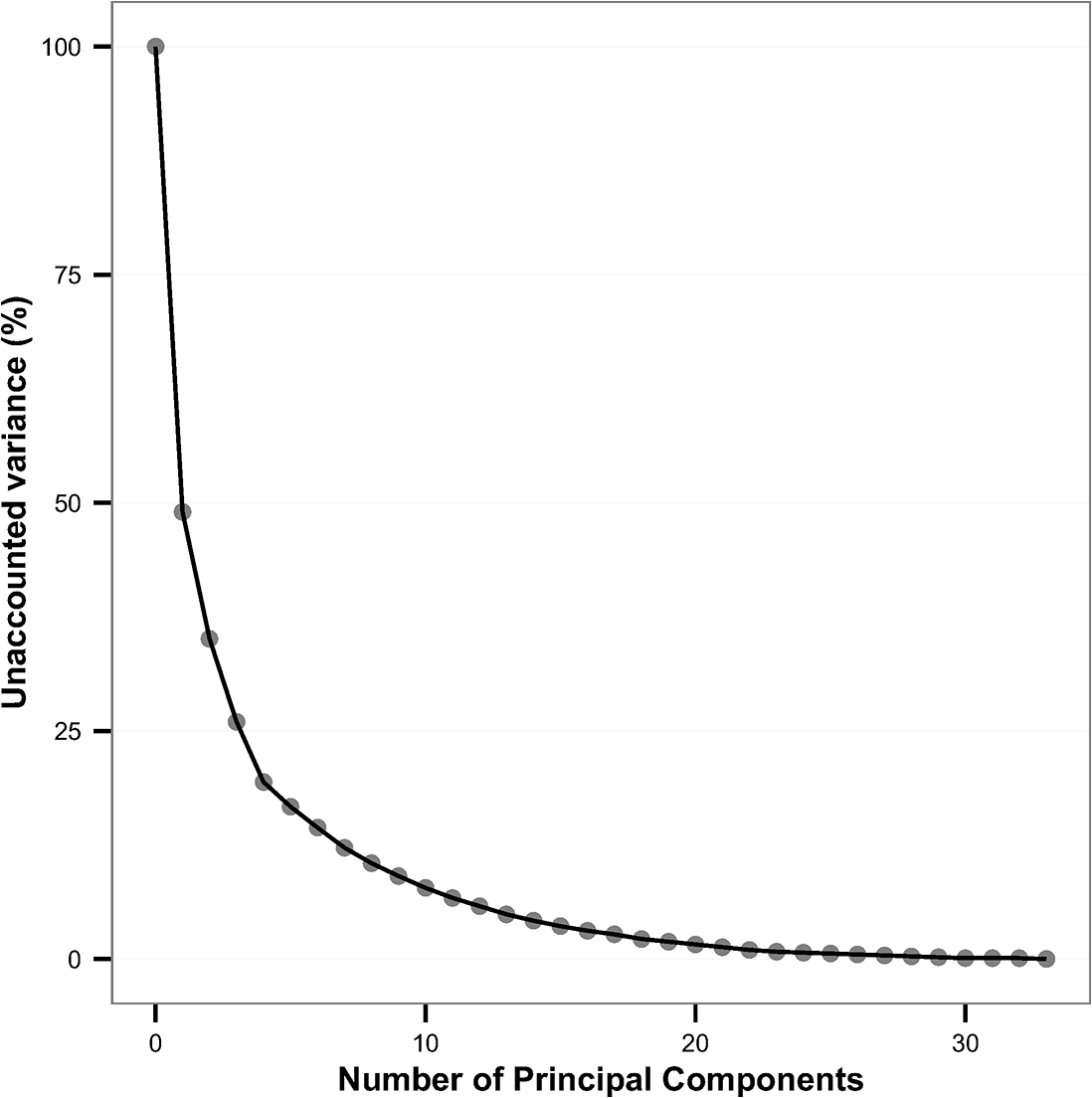
Proportion of unaccounted variance against the number of PC included. Scree plot showing the proportion of variance that is unaccounted for against the number of Principal Components included. The first four components derived from Principal Component Analysis account for over 80% of the variance in the original data.

PCA was conducted using the variables representing the time spent in the various behaviour categories during a given hour averaged over the study period: standing, lying and active for each of the 24 hours. As noted, the number of steps per hour was highly correlated with the proportion of time spent in the active behaviour category and so this variable was dropped from the analyses. Fig 2 illustrates a scree plot which describes the decreasing rate at which the variance in the overall data can be accounted by including additional Principal Components (PCs). The first PC can account for 51% of the variance, while the first four PCs taken together account for just over 80% of the variance in the data.

**Fig 3:**
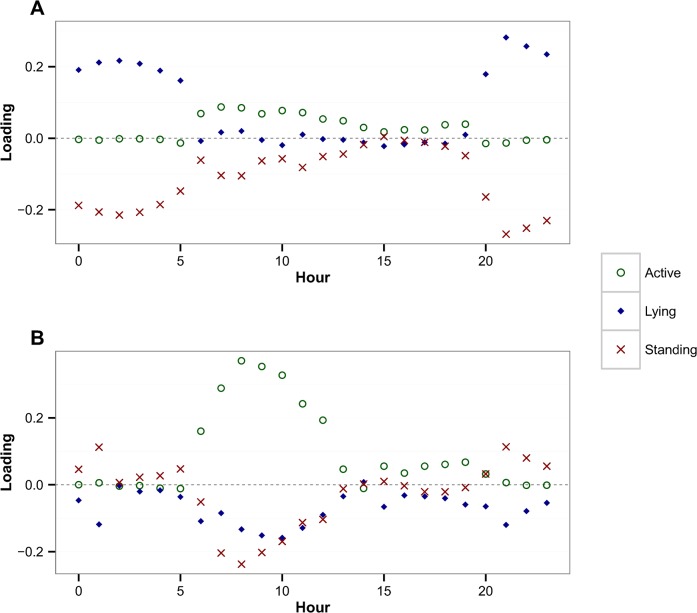
Loading value for each behaviour across 24 hours. The loading of each cattle behaviour variable, active (green hollow circles), lying (blue diamonds), and standing (red crosses) across 0:00 to 23:00 hours, on [A] PC1 and [B] PC2. These figures indicate that much of the heterogeneity in cattle behaviour during the night and day time is accounted for by PC1 and PC2, respectively.

The loadings of variables representing each behaviour in a given hour are shown for PC1 (Fig 3A) and PC2 (Fig 3B), respectively. Fig 3A indicates that variables representing the percentage of time spent lying and standing between 20:00 hrs and 5:00 hrs are highly correlated, positively and negatively respectively, with the first principal component (PC1). Fig 3B indicates that the variables representing the percentage of time spent on different behaviours between 6:00 hrs and 12:00 hrs are strongly correlated with PC2. These two figures suggest that there is substantial variability in cattle behaviour during the night and early morning, which can be accounted for by PC1 and PC2, respectively. The loading plots for PC3 and PC4 indicated that behaviours between 5:00 hrs and 7:00 hrs and those between 8:00 hrs and 16:00 hrs were strongly associated with changes in the values of PC3 and PC4, respectively (Figs A and B in S1 Fig).

**Fig 4:**
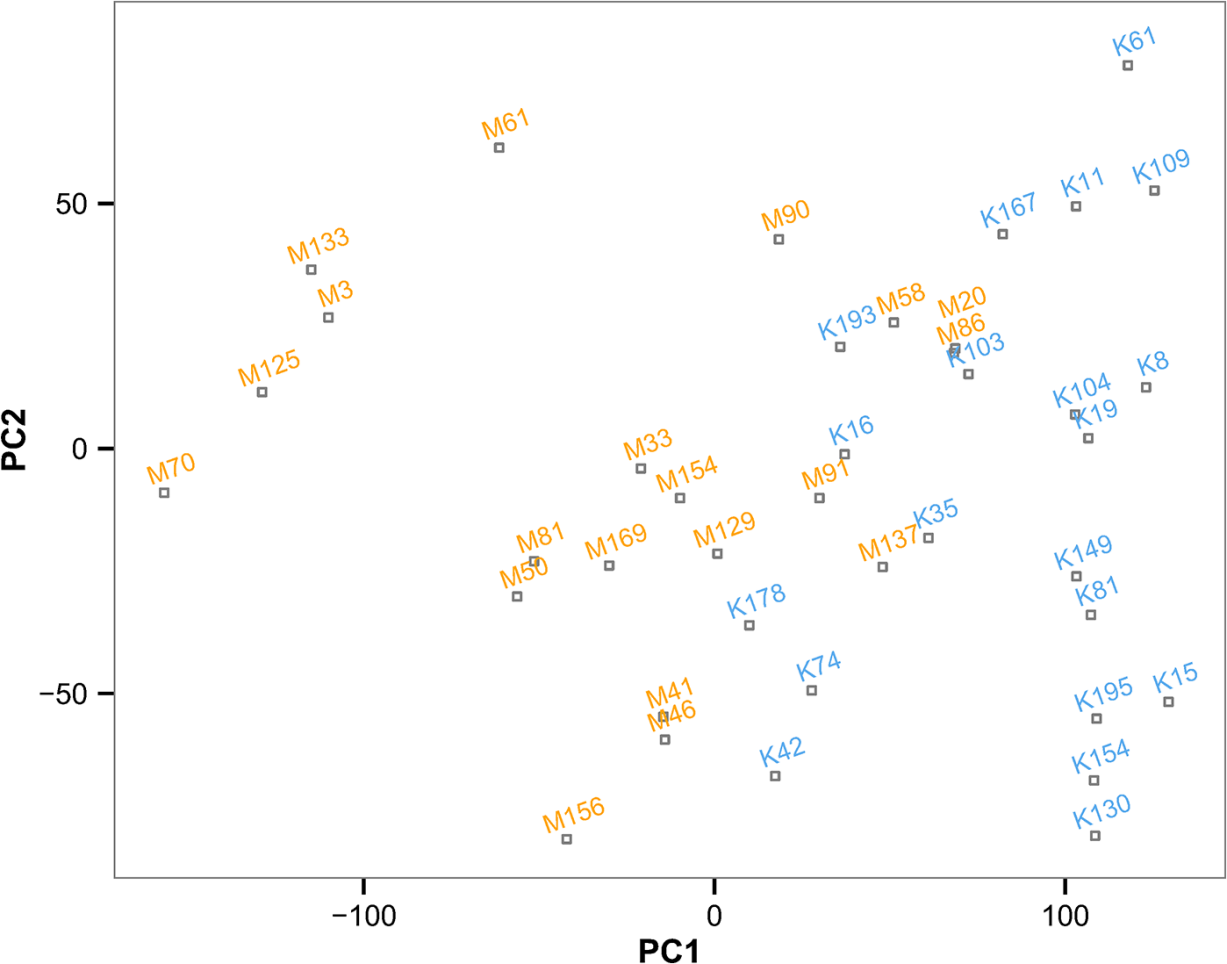
Scatter plot illustrating the location of each animal in PC1 and PC2 space. Annotations of plots represent each of 40 individual cattle from the Kasero (blue) and Makale (orange) veterinary camps of Petauke District in the Eastern Province of Zambia. PC1 and PC2 accounted for 51.0% and 14.1% of the total variance in the data.

The PCA also reports the score of each principal component for all observations. A useful two-dimensional configuration for viewing the data is therefore often provided by plotting observations in the first two PCs. In this case the first and second PCs accounted, respectively, for 51.0% and 14.1% of the overall variability in the data. Fig 4 plots the location of each animal based on their scores in PC1 and PC2. A clear trend can be observed in that animals from Kasero have high positive values in PC1 with a relatively narrow range of values among animals. On the other hand, those from Makale tend to have lower PC1 values, with more variation among animals. This indicates that animals from Kasero spent more time lying down between the hours of 20:00 and 5:00 than did those from Makale. However, there seems no clear difference in the values of PC2 between vet camps. This would suggest that the large variance in different behaviours between 6:00 and 12:00 hrs can be attributed to individual animals and does not reflect vet camp-specific characteristics. Table 1 shows the ICC value, its 95% Confidence Interval (CI) and *p* value indicating the presence of clustering within vet camp for each PC score obtained using analysis of variance (ANOVA). There was a significant clustering effect in the scores for PC1 and PC3. However, the scores for PC2 and PC4 were not different between vet camps.

**Table 1:** Intraclass correlation coefficient within vet camp obtained from the analysis of variance.

Components	ICC within vet camp (95% CI)	*p* value
PC1	0.67 (0.04–1.0)	p <0.001
PC2	0.00 (0.00–0.14)	p = 0.71
PC3	0.15 (0.00–0.61)	p = 0.04
PC4	0.00 (0.00–0.14)	p = 0.91

The degree to which PC score of individual animal within a vet camp was similar was examined by calculating the intraclass correlation coefficient (ICC) and its 95% confidence interval for the first four PCs. The one-sided *F* test was conducted to examine if the derived ICCs were significantly different from 0. The score on PC1 and PC3 for individual animals were more similar within vet camps and there were greater variations between animals in different camps.

**Fig 5:**
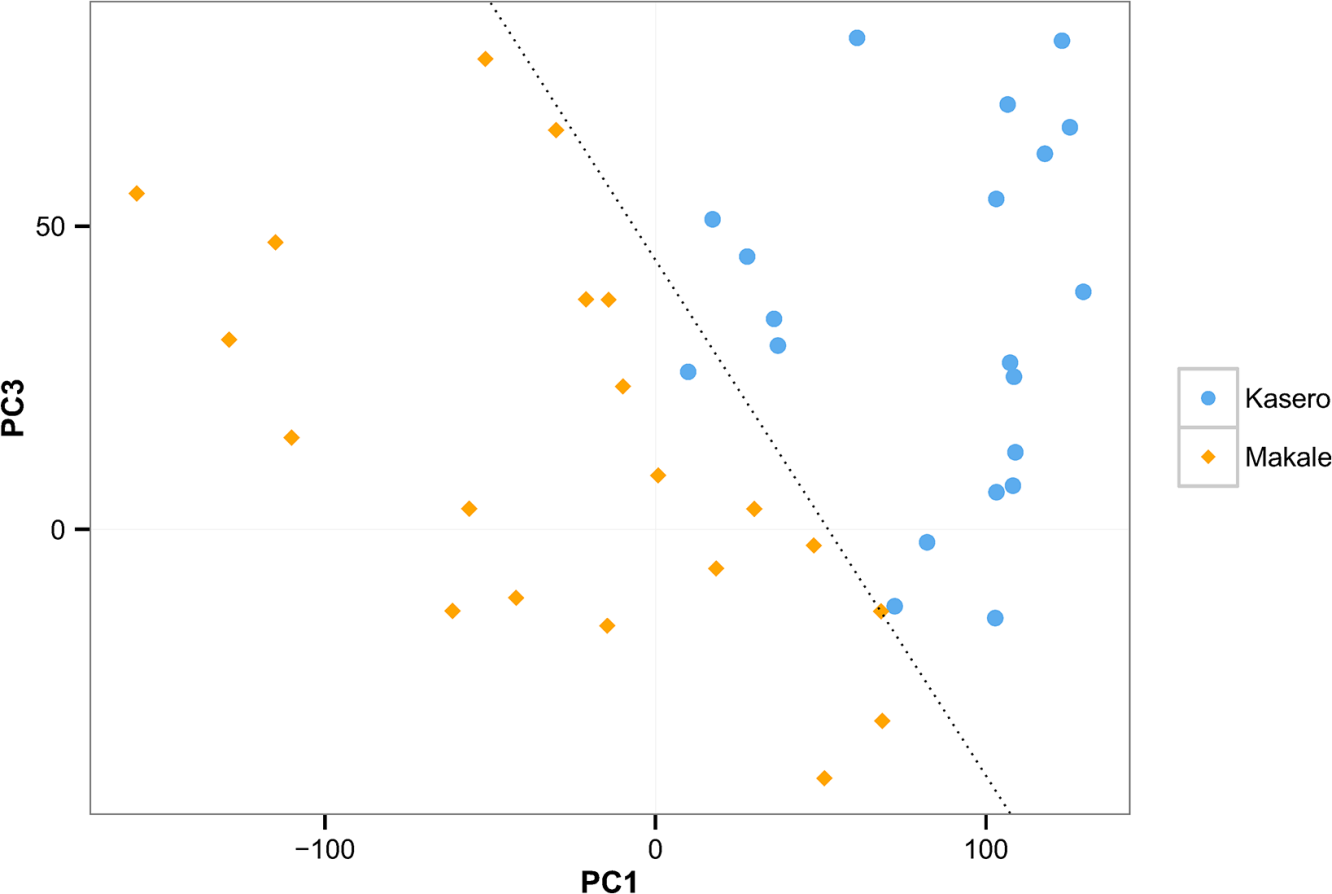
Scatter plot of animals in PC1 and PC3 space. Each point located in PC1 and PC3 space, represents individual cattle from the Kasero (blue) and Makale (orange) veterinary camps of Petauke District in the Eastern Province of Zambia. The dotted line represents a classification line predicted from a logistic regression model that best separates the animals from the two vet camps. PC1 and PC3 accounted for 51.0% and 9.0% of the total variance in the data.

The scores on the third, fourth and succeeding components may also need to be considered if the effective dimensionality is greater than two [18]. PC3 and PC4 accounted for 9.0% and 6.6% of variance, respectively, and should therefore not be ignored; their use being dependent on the question of interest. For example, it can be seen from the ICC values reported in Table 1 that both PC1 and PC3 appear to capture between-vet camp variation, something not seen for PC2 and PC4 which may capture individual-based variation. Fig 5 shows a plot of all cattle based on their scores in PC1 and PC3. It can be seen from this figure that the combination of PC1 and PC3 can clearly separate the animals from each of the two vet camps. The predicted class separation using a logistic regression model based on PC1 and PC3 (dotted line in Fig 5; the results of a logistic regression can be found in S1 Table) indicates almost total success in separating the two vet camps, with only one misclassified animal from the Kasero vet camp. However, PC2 was found to be non-informative in any attempt to separate animals from the two vet camps, nor did other PCs improve classification accuracy.

**Fig 6:**
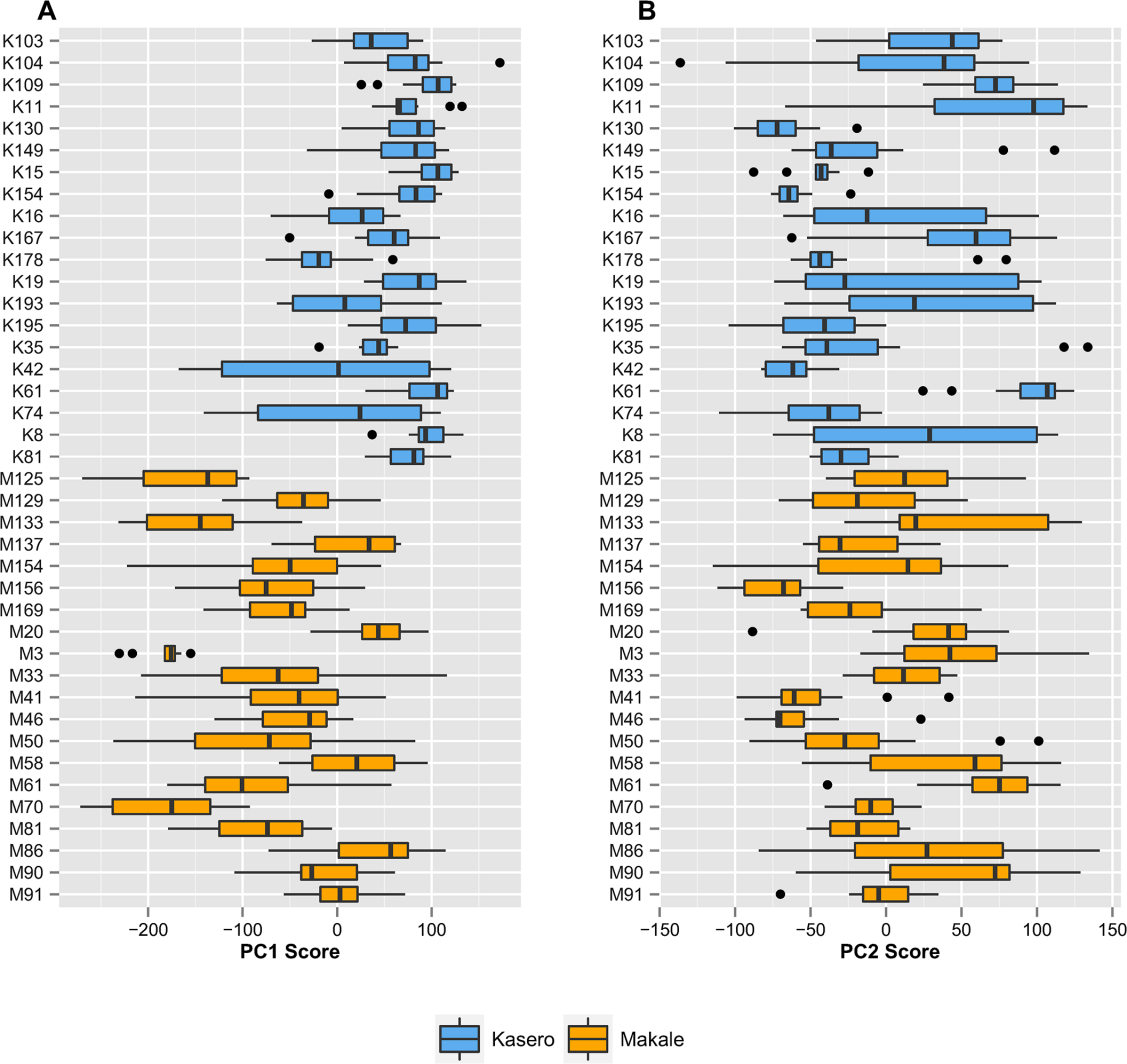
Box-plot showing within-animal daily variations of PC scores. Daily PC scores over the study period were calculated for 40 cattle from Kasero (blue) and Makale (orange) veterinary camps and their daily variations are shown for [A] PC1 and [B] PC2. This calculation was based on the loading values of behaviour variables in a given hour, which were assumed to be consistent over the studied period, and the proportion of time each individual spent in any of the three behaviours in the hour in a given day.

As described above, to aid in the application and interpretation of the PCA, the mean percentage of times spent on standing, lying and active behaviours across each hour were averaged over the period for which observations were available (13 or 14 days). Thus the variables used in the PCA did not account for daily variation in behaviour within individual animals. To validate the use of mean percentages for each variable, averaged over the study period, the within-animal and between-animal variations in PC values were investigated. Assuming that the obtained loading values would tend to be consistent over the study period, scores for PC1 to PC4 of each animal for each day were calculated using these loading values together with the proportion of time an animal spent in each type of behaviour. Fig 6A shows the daily variation of scores for PC1 across animals over the study period. With the exception of two animals the within-animal variation in PC1 for cattle from Kasero appears to be smaller in comparison to animals from Makale; the overall values also tend to be higher. On the other hand, the variation in PC2 (shown in Fig 6B), demonstrates no such clear vet camp-specific trends. Given the fact that there were differences between vet camps in the values of PCs, the clustering of scores on each PC within animals was also investigated using ANOVA for each vet camp. Table 2 summarises the ICC value and its 95% CI for each component in Kasero and Makale. Moderate ICC values across the primary PCs in both vet camps suggest that the correlation between cattle behaviour on any given pair of days is not negligible; a PCA based on daily cattle behaviour would therefore likely lead to unnecessary redundancy in the data variance. Therefore, the use of variables averaged over the study period is a reasonable method for summarising the data.

**Table 2:** Intraclass correlation coefficients within animal on the first four PCs.

Vet camp	Kasero	Makale
Components	ICC within animal (95% CI)	ICC within animal (95% CI)
PC1	0.34 (0.17–0.51)	0.53 (0.35–0.71)
PC2	0.44 (0.26–0.63)	0.39 (0.21–0.57)
PC3	0.37 (0.19–0.54)	0.28 (0.12–0.47)
PC4	0.26 (0.10–0.41)	0.25 (0.10–0.41)

Intraclass correlation coefficients for PC scores over the studied period within individual animal were calculated separately in each of two vet camps. Moderate to high ICC values for the first four PCs indicate that there would be little benefit in employing daily behaviour variables, at the cost of increasing the total variance in the data. All ICC values were significantly different from 0 (*p* < 0.001).

## Discussion

The results of this study clearly indicate that motion sensors can be used to study cattle movement behaviour under a traditional African livestock management regime and can identify important patterns. The technology was accepted by both the farmers and their cattle. The farmers may have accepted the technology to be used on their animals more readily due to the fact that they were fully informed of what was happening from the beginning. It has been observed that when people understand a control option and its potential benefits, they are more likely to respond positively [19]. Animals appeared not to mind the attachment of motion sensors to their hind legs. As a precaution in trying to prevent too much discomfort, the Velcro straps attaching the sensors were not attached too tightly around the animals’ legs and the sensors were changed to the other hind leg after one week of attachment.

Motion sensors have been successfully used or evaluated as tools to monitor and classify animal behaviour by other researchers [20–23]. Despite being used during adverse wet and muddy weather conditions in the rainy season, the motion sensors recorded the standing, active and lying down behaviours as well as step counts of cattle with minimal labour and caused no major technical problems during the data capture phase. Although very high correlation was found between estimation of the number of steps taken and the time spent on active behaviour in a given hour, there were some outlying cases where poor agreement was observed. These were largely confined to hours during which animals spent more than 50% of their time in standing behaviour and the tendency was to underestimate the number of steps, relative to the expected number based on linear regression. This would suggest that while animals are standing they may make some movement (i.e. the animal was deemed to be "active") but that this will not result in a full step being recorded. Underestimation in the number of steps is less likely to occur when the animals are lying down for the majority of the time period and this was borne out in the data. Traditional visual observational techniques of cattle movement, such as constant video surveillance, allows for non-invasive behaviour monitoring, but logging the movement patterns on individual animals over long periods of time demands a large amount of labour, equipment, and time [1, 24, 25]. The use of motion sensor technology in the study of cattle movement behaviour is relatively new and was successfully used in this study to collect movement data on traditionally managed Angoni cattle in sub-Saharan Africa.

One of the challenges in dealing with the reasonably large sets of data that come from the use of such devices in the field is finding appropriate methods to summarise and interpret these data. Principal components analysis was used in this study to characterise cattle movement and to explore which of the various behaviours, and what times of the day, were important in determining overall variability in movement behaviour. The original 72 dimensional data set was reduced using PCA to four variables which accounted for over 80% of the variability in the data. In particular the first PC accounted for more than half of total variance while the loading plot of variables on PC1 indicated that the majority of variability in cattle behaviour existed in standing and lying behaviours during the night time, and that this varied markedly between the Kasero and Makale vet camps. In contrast to PC1, there was no marked difference in PC2 score values between the two vet camps and a wide variation in behaviour patterns during the morning was observed across all of the cattle in the study, irrespective of vet camp. Thus, it could be assumed that behaviour of animals between 7:00 and 12:00 hrs is associated with factors residing at the individual animal level and may be useful when investigating issues such as varying health status.

The results of a logistic regression model indicated that the inclusion of PC1 and PC3 was enough to separate animals almost perfectly into two vet camps, further supported the view that there are substantial differences in the lying and standing behaviour of cattle during the night and in the early morning in these two vet camps. Since an objective of future research is to explore the relationships between animal health status and their behaviour, a better understanding of the causes for the observed differences in movement behaviour between the two vet camps is important. One possible explanation for this could be the slight difference in time of year at which these studies were conducted in each vet camp. Due to limitations on the number of motion sensors that were available, the study was initially carried out in Kasero before being repeated in Makale. The rains had started during the study in Kasero but intensified as the study moved to Makale. Kraals where cattle are kept for long periods of time, especially in the rainy season, become heavily contaminated with dung and mud [26]. In our study, Makale had received more rains and this may have led to deterioration in the condition of the ground, to the point of unsuitability for lying and may, at least in part, explain why animals were standing more in that particular vet camp. It is known that cattle lying behaviour can be used as a measure of well-being in the dairy industry, where large increases in standing time have been observed to be associated with increasing severity of lameness [27]. Overtly lame animals were not observed in the current study, although a detailed clinical examination of the condition of these animals’ feet were not conducted and a more general investigation of possible association between kraal status and the lying behaviour of animals would be justified.

Movement behaviour was found to vary greatly within an individual over a number of days, as well as among different animals. Although PC1 was found to be significantly affected by unknown vet camp-related factors, heterogeneity in daily variation among cattle was observed for all the primary PCs. This implies that some animal-level factors may cause daily variation in cattle behaviour. More detailed study is required to unravel the likely causes of differences in cattle behaviour between animals and across days.

Previous studies in the two veterinary camps have shown that Makale has a high prevalence of trypanosomiasis (*T*. *congolense*, savannah type) while Kasero has a high prevalence of theileriosis caused by *T*. *parva* [12, 13]. Infection with these and other common pathogens would cause a variety of clinical signs, including various degrees of anaemia. The potential value of information relating the disease status of cattle and animal behaviour is well recognised [28]. We will therefore investigate associations between disease status in animals, haemoglobin levels and their movement behaviour in a subsequent paper.

In conclusion, the use of motion sensors as a tool for the monitoring and evaluation of cattle movement behaviour in rural Zambia was validated in this study. The study also provides a baseline for future research on traditionally managed cattle movement behaviour in sub-Saharan Africa. Moreover, the use of PCA successfully reduced the dimensionality of the data from 72 original variables to a manageable set of just four principal components. PCA also highlighted that the marked differences in standing/lying behaviour during the night and active behaviour in the morning were due to strong vet camp and individual animal-level factors, respectively. Future studies will focus on elucidating those factors, with a particular emphasis on investigating the relationship between animal behaviour and compromised health status due to infectious diseases such as theileriosis and trypanosomiasis.

## Supporting Information

S1 Dataset Data contains the behaviour of 40 cattle in a given hour over the study period. (CSV)

S1 Fig Loading value for each behaviour across 24 hours for [A] PC3 and [B] PC4. (PDF)

S1 Table Results of logistic regression to explain the binary vet camp identity by PCs. (DOCX)
