# Supplementary figures and images for: Movement Behaviour of Traditionally Managed Cattle in the Eastern Province of Zambia Captured Using Two-Dimensional Motion Sensors
Source: PLoS One. 2015 Sep 14;10(9):e0138125. doi: 10.1371/journal.pone.0138125 (PMC4569424; doi:10.1371/journal.pone.0138125)

**A**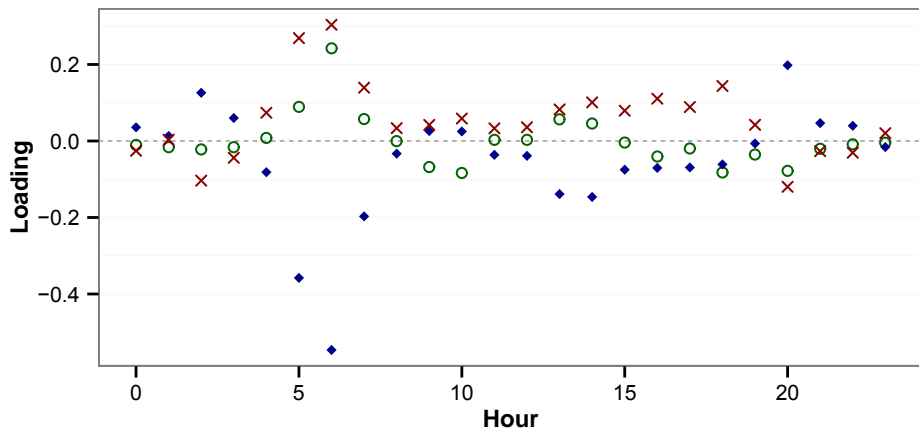**B**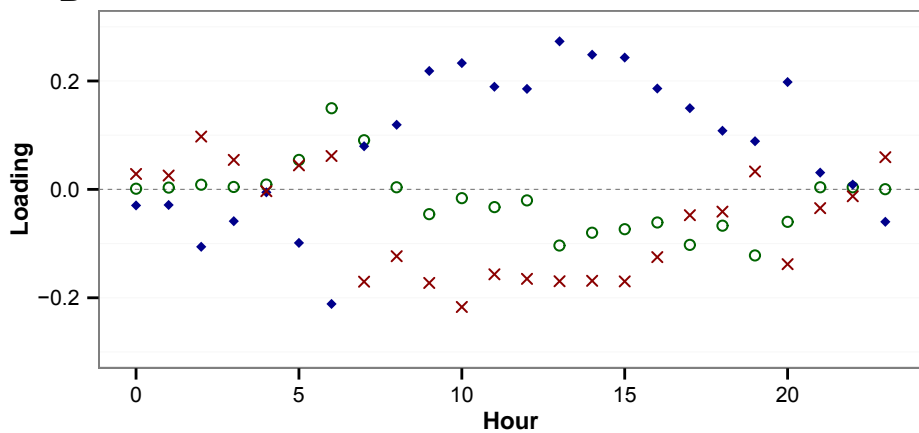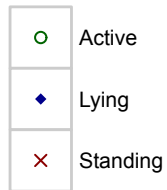

Supplement: S1 Fig — (PDF) [file pone.0138125.s002.pdf]
