# Supplementary material for: Movement Behaviour of Traditionally Managed Cattle in the Eastern Province of Zambia Captured Using Two-Dimensional Motion Sensors
Source: PLoS One. 2015 Sep 14;10(9):e0138125. doi: 10.1371/journal.pone.0138125 (PMC4569424; doi:10.1371/journal.pone.0138125)
Supplement: S1 Table — (DOCX) [file pone.0138125.s003.docx]

**S1 Table**

Results of a multivariable logistic regression model to predict the vet camp identity (Kasero = 0 and Makale = 1) based on Principle Component (PC) values. Only PC1 and PC3 were found to be associated with the vet camp identity; which led to the following logistic regression model:

glm (vet_camp ~ PC1 + PC3, family = “binomial”)

|  |  |  |  |  |  |  |
| --- | --- | --- | --- | --- | --- | --- |
|  |  |  |  | 95%CI | |  |
| Covariate |  | Coefficient |  | Lower | Upper | p-value |
|  |  |  |  |  |  |  |
| Intercept |  | 8.80 |  | 0.03 | 17.6 | 0.05 |
|  |  |  |  |  |  |  |
| PC1 |  | -0.169 |  | -0.34 | 0.00 | 0.05 |
|  |  |  |  |  |  |  |
| PC3 |  | -0.198 |  | -0.42 | 0.02 | 0.07 |
